# HEALTH CARE SETTINGS: A MISSED OPPORTUNITY TO ADDRESS HEARING LOSS

**DOI:** 10.1093/geroni/igad104.0531

**Published:** 2023-12-21

**Authors:** Margaret Wallhagen

**Affiliations:** University of California, San Francisco, San Francisco, California, United States

## Abstract

**Background:**

Identifying hearing loss (HL) within the healthcare system is essential to mitigate its risk for associated negative psychosocial and health conditions, including isolation and depression yet is rarely assessed. This qualitative study sought to gain in-depth understandings of how HL affects communication regarding care preferences within the older adult-partner dyad in the context of chronic illness. Its premise was that older adults with HL may not be able to participate effectively in care discussions and are at risk of not receiving patient centered care. Method: Planned as in-person, pandemic restrictions mandated transition to zoom. 15 older adults (≥60) with HL and 13 partners participated.

**Results:**

One key story line evolving from the data is “Compulsory Self-Advocacy”. Almost uniformly, participants reported that healthcare practitioners “Never Asked” about HL. During one-on-one communication, HL wasn’t necessarily brought to the surface and often went unnoted because participants didn’t see it as pertinent to issues discussed. Still, they were unsure the practitioner was aware of their HL. Rather, they asked that information be repeated or noted they had trouble hearing or understanding when they realized problems. Reporting their HL didn’t generally result in on-going consideration of their specific communication needs. They had to self-advocate to obtain accurate information or bring their partner to appointments to help. Some commented on the value of policies mandating access to notes or written reports.

**Conclusions:**

Healthcare settings provide unique opportunities to identify HL and initiate interventions that may mitigate its negative effects, including social isolation and loneliness.

